# Diffusion–Based Virtual MR Elastography of the Liver: Can It Be Extended beyond Liver Fibrosis?

**DOI:** 10.3390/jcm10194553

**Published:** 2021-09-30

**Authors:** Takashi Ota, Masatoshi Hori, Denis Le Bihan, Hideyuki Fukui, Hiromitsu Onishi, Atsushi Nakamoto, Takahiro Tsuboyama, Mitsuaki Tatsumi, Kazuya Ogawa, Noriyuki Tomiyama

**Affiliations:** 1 Department of Diagnostic and Interventional Radiology, Osaka University Graduate School of Medicine, Suita 565-0871, Japan; horimsts@med.kobe-u.ac.jp (M.H.); fukui-hide@radiol.med.osaka-u.ac.jp (H.F.); h-onishi@radiol.med.osaka-u.ac.jp (H.O.); a-nakamoto@radiol.med.osaka-u.ac.jp (A.N.); ttsuboyama@gmail.com (T.T.); m-tatsumi@radiol.med.osaka-u.ac.jp (M.T.); ogawa-kazu@radiol.med.osaka-u.ac.jp (K.O.); tomiyama@radiol.med.osaka-u.ac.jp (N.T.); 2 Department of Radiology, Kobe University Graduate School of Medicine, Kobe 650-0017, Japan; 3 NeuroSpin, CEA-Saclay, Paris-Saclay University, 91191 Saclay, France; denis.lebihan@gmail.com; 4 National Institute for Physiological Sciences (NIPS), Okazaki 444-8585, Japan; 5 Human Brain Research Center, Graduate School of Medicine, Kyoto University, Kyoto 606-8303, Japan

**Keywords:** diffusion-weighted imaging, MR elastography, virtual MR elastography, liver, hepatocellular carcinoma, metastatic liver cancer

## Abstract

*Background*: Strong correlation has been reported between tissue water diffusivity and tissue elasticity in the liver. The purpose of this study is to explore the capability of diffusion–based virtual MR elastography (VMRE) in the characterization of liver tumors by extending beyond liver fibrosis assessments. *Methods*: Fifty-four patients (56 liver tumors: hepatocellular carcinoma (HCC), 31; metastases, 25) who underwent MRE, diffusion-weighted imaging (DWI) (*b*: 0, 800 s/mm^2^), and VMRE (*b*: 200, 1500 s/mm^2^) were enrolled. The MRE shear modulus (µ_MRE_), apparent diffusion coefficient (ADC), and shifted ADC (sADC) were obtained. Virtual stiffness (µ_diff_) was estimated from the relationship between µ_MRE_ and sADC. A linear discriminant analysis combining VMRE and MRE to classify HCC and metastases was performed in a training cohort (thirty-two patients) to estimate a classifier (C), and evaluate its accuracy in a testing cohort (twenty-two patients). Pearson’s correlations between µ_MRE_, sADC, and ADC were evaluated. In addition to the discriminant analysis, a receiver operating characteristic (ROC) curve was used to assess the discrimination capability between HCC and metastases. *Results*: The correlations between µ_MRE_ and sADC were significant for liver, HCC, and metastases (*r* = 0.91, 0.68, 0.71; all *p* < 0.05). Those between µ_MRE_ and ADC were weaker and significant only for metastases (*r* = 0.17, 0.20, 0.55). µ_diff_ values were not significantly different between HCC and metastases (*p* = 0.56). Areas under the curves (AUC) to differentiate HCC from metastases were as follows: VMRE, 0.46; MRE alone, 0.89; MRE + VMRE, 0.96. The classifier C also provided better performance than MRE alone, in terms of sensitivity (100 vs. 93.5%, respectively) and specificity (92 vs. 76%, respectively, *p* = 0.046). *Conclusions*: The correlation between sADC and µ_MRE_ was strong both in the liver and in tumors. However, VMRE alone could not classify HCC and metastases. The combination of MRE and VMRE, however, allowed discriminant performance between HCC and metastases.

## 1. Introduction

A strong correlation has been recently reported between tissue water diffusivity and tissue elasticity in the liver [1,2]. Specifically, the shifted apparent diffusion coefficient (sADC) values (obtained from the *b* values of 200 and 1500 s/mm^2^) in liver parenchyma were proven to be strongly correlated with the liver tissue elasticity obtained with MR elastography (MRE) in a small cohort (*n* = 15) [1], and in a larger patient cohort (*n* = 74) [2]. In both studies, tissue stiffness generated from sADC values was obtained, and was graded accurately depending on the stage of liver fibrosis. These findings suggested that diffusion-weighted imaging (DWI)-based or Intravoxel Incoherent Motion imaging (IVIM)-based virtual elastography (VMRE) [1] could serve as an alternative to MRE for the staging assessment of liver fibrosis. Additionally, tissue elasticity measurements are reportedly useful in the characterization of liver tumors [3,4,5]. Malignant tumors have greater cellularity, and may result in increased stiffness that can be evaluated with MRE [4]. Thus, VMRE may also be useful for liver tumor characterization. However, the existence of a relationship between water diffusivity and tissue elasticity in other than liver parenchyma is still unknown.

Liver cancers constitute the fifth leading cause of cancer-related deaths worldwide [6]. Hepatocellular carcinoma (HCC) accounts for the majority of primary liver cancers. Secondary liver cancer results from malignant tumors of extrahepatic origin that metastasize to the liver. In this way, HCC and metastatic liver cancer (metastases) account for a large part of liver malignancies. Differentiation of HCC from metastases is clinically important because the differences in tumor characteristics imply different therapeutic strategies. The differentiation of HCC from metastases is usually based on tumor enhancement patterns observed after contrast injection in combination with the patient’s clinical history (i.e., presence of chronic liver disease, past cancer history, and serum tumor markers). HCC typically exhibits arterial hyperenhancement, nonperipheral washout, and enhancing capsule (the Liver Imaging Reporting and Data System (LI-RADS) version 2018), whereas metastases exhibit hypovascular and rim-like enhancements [7,8]. However, contrast materials are contraindicated for some patients and sometimes cause side effects [9]. Therefore, the availability of an accurate method that does not require contrast agents would be beneficial for many patients.

Previous studies have shown that MRE is more accurate than standard DWI (based on ADC values obtained with *b* values < 1000 s/mm^2^) for differentiating benign and malignant liver tumors [4]. However, it is expected that sADC values (and their conversion to tumor elasticity) might perform better to help differentiate liver tumors. The purpose of this study was the exploration of the capability of sADC-based VMREs for liver tumor characterization by extending beyond liver fibrosis assessments. Specifically, we focused on the value of sADC-based VMREs in differentiating HCCs from metastases.

## 2. Materials and Methods

This retrospective study was approved by the institutional review board of our hospital that waived the requirement for informed consent.

### 2.1. Patient Population

**Figure 1:**
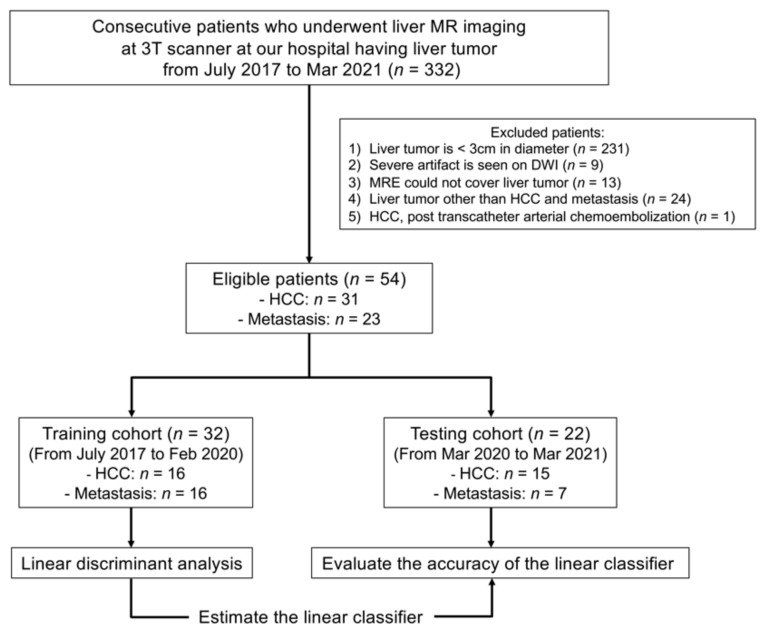
Flowchart of the patient enrollment process.

Between July 2017 and March 2021, 332 liver tumor patients underwent liver MR imaging, including MRE, DWI (*b* values, 0 and 800 s/mm^2^), and DWI-based VMRE (*b* values, 200 and 1500 s/mm^2^) at our hospital. We excluded patients who met the following exclusion criteria: (a) tumor diameter < 3 cm (*n* = 231), (b) severe artifacts on DWI (*n* = 9), (c) tumors outside (*n* = 9) or inside the cross-hatched area in the stiffness map in MRE (*n* = 4), (d) liver tumor other than HCC and metastases (*n* = 24), and (e) HCC treated by transcatheter arterial chemoembolization (*n* = 1). The limit on tumor size (3 cm) was set based on the limited spatial resolution of MRE and the presence of artifacts. Below 3 cm tumors are not masses, but nodules which are hard to detect with standard MRE, hence nodules were excluded. In total, the final study population included 54 patients (mean ± standard deviation age: 69.4 ± 9.3 years, range: 47–87 years, HCC: *n* = 31, metastases: *n* = 23) (Figure 1, Table 1). Out of the 54 patients, 32 patients (from July 2017 to February 2020, HCC: *n* = 16, metastases: *n* = 16) were randomly assigned to define a training cohort, and the remaining 22 patients (from March 2020 to March 2021, HCC: *n* = 15, metastases: *n* = 7) were assigned to a testing cohort to assess the performance of a linear discriminant analysis based on its capacity to differentiate HCC from metastases. VMRE was also evaluated in the liver parenchymas of 45 of these patients (we could not calculate the liver parenchymas in nine patient cases because of severe artifacts on the DWI in the parenchyma). MR images were analyzed by two radiologists (board-certified with nine years of experience each in abdominal imaging) who were blinded to the diagnosis.

### 2.2. MR Imaging Acquisition and Analysis

Thirty-three patients were examined on a 3.0 T MR system (Discovery 750, GE Healthcare, Milwaukee, WI, USA) with a 32-channel cardiac phased-array coil. Sixteen patients were examined on a 3.0 T MR system (Signa Architect, GE Healthcare, Milwaukee, WI, USA) with a 30-channel adaptive imaging receive (AIR) coil, and five patients were examined on a 3.0 T MR system (Discovery 750 w, GE Healthcare, Milwaukee, WI, USA) with a 32-channel cardiac phased-array coil. Scan parameters are summarized in Table 2.

### 2.3. MR Elastography

For MRE, mechanical vibration (60 Hz) to the liver was transmitted from a passive driver that was connected to an active driver. The passive driver was positioned on the right chest wall of the patient (placed in a supine position) [10]. An active driver was located outside the imaging room. It generated a pneumatic vibration that was transmitted to the passive driver through a plastic tube. The MRE was performed with a two-dimensional echo-planar sequence, which acquired a set of four 10 mm axial slices which covered a slab of 52 mm (below the subphrenic level to a level above the gallbladder of the liver). Stiffness maps were created with an inversion algorithm, and the shear modulus (µ_MRE_) was calculated from regions-of-interest (ROI) placed on the stiffness maps [10]. The confidence map is a statistical derivation used to overlay a “cross-hatched” on the stiffness map to exclude regions in the liver that have less reliable (i.e., noisy and discontinuous) stiffness data. We placed ROI avoiding such cross-hatched area on the confidence map.

### 2.4. Diffusion MRI

For DWI acquisition, a fat-suppressed, spin-echo echo-planar sequence was used (*b* values of 0 and 800 s/mm^2^ with respiratory triggering for the ADC, and 200 and 1500 s/mm^2^ with breathholding for the sADC, on 7 mm axial slices). Diffusion-sensitizing gradient pulses were simultaneously applied on the x-, y-, and z-axes. Scan parameters are summarized in Table 2. ADC and sADC values were calculated from the DWI values from the ROIs placed on the liver tumor, as follows,

ADC (mm^2^/s) = ln(S_0_/S_800_)/800 (1)

sADC (mm^2^/s) = ln(S_200_/S_1500_)/1300 (2)

From the sADC values, the DWI-based shear modulus (µ_diff_) of the tumor was calculated as in the previous report [1],

µ_diff_ (kPa) = α ln(S_200_/S_1500_) + β (3)

where α and β are calibration coefficients. These calibration coefficients were estimated in our patient population for the liver parenchyma, HCC, metastases, and malignant tumors (HCC and metastases) based on a linear regression between the µ_MRE_ and sADC values.

### 2.5. Image Analysis

**Figure 2:**
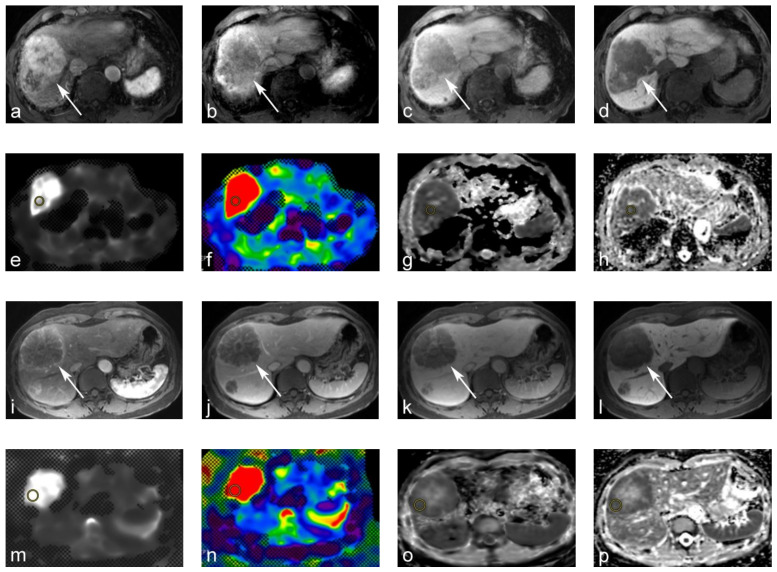
69-year-old male with moderately differentiated hepatocellular carcinoma (HCC). (**a**–**d**) Gd-EOB-DTPA-enhanced magnetic resonance image (MRI): (**a**) tumors show arterial hyperenhancement on the arterial-phase. (**b**,**c**) Tumor shows a washout on the portal venous and transitional phase; (**d**) tumor shows a hypointensity on the hepatobiliary phase. (**e**,**f**) MR elastography (MRE). The tumor yielded a stiffness of 10.71 kPa on the MRE. (**g**,**h**) Apparent diffusion coefficient (ADC) maps. The tumor yielded a value of 0.63 × 10^−3^ mm^2^/s on the shifted ADC (sADC) map, and a value of 0.93 × 10^−3^ mm^2^/s on the ADC map. 60-year-old male with metastatic hepatic cancer from colorectal cancer. (**i**–**l**) Gd-EOB-DTPA-enhanced MRI. (**i**,**j**) Tumor showed rim-like enhancements on the arterial and portal venous phase. (**k**,**l**) The tumor was hypointense on the transitional and hepatobiliary phases. (**m**,**n**) MRE. The tumor yielded a stiffness of 15.59 kPa on MRE. (**o**,**p**) ADC maps. The tumor also yielded an sADC value of 0.72 × 10^−3^ mm^2^/s on the sADC map, and 0.86 × 10^−3^ mm^2^/s on the ADC map.

Two radiologists placed the ROIs independently on the stiffness, sADC, and ADC maps. The ROIs were placed on the slices in which the tumors had maximum diameters. Care was taken to place ROIs on the same region as those on the stiffness and ADC/sADC maps, and to avoid necrotic, bleeding, or inhomogeneous areas (Figure 2). ROIs were also placed on the homogeneous area on the right lobe of the liver parenchyma on these maps for 45 patients (nine patients had severe artifacts in this part of the parenchyma).

### 2.6. Statistical Analysis

The inter-reader reliability was evaluated based on the calculation of interclass correlation coefficients. Correlations between sADC, ADC, and the MRE shear modulus (µ_MRE_) were evaluated based on Pearson’s correlation coefficients. The mean sADC and ADC, mean µ_MRE_, and µ_diff_ values in the HCC and metastases were compared using a Student’s *t*-test. Receiver operating characteristic (ROC) curves and discriminant analyses were conducted to assess the discrimination capability between HCC and metastases, (a) sADC alone, (b) ADC alone, (c) MRE alone, (d) combinations of MRE and sADC, and (e) combinations of MRE and ADC. Finally, a linear discriminant analysis between the HCC and the metastases was performed in a training cohort to predict the discriminant classifier based on the sADC and MRE values, and to evaluate its performance in a testing cohort. The differences between classifier and MRE values in sensitivities, specificities, and accuracies were compared using the McNemar’s test. *p*-values < 0.05 were considered statistically significant.

Statistical analyses were performed with the use of SPSS (version 24, IBM, Armonk, NY, USA), and discriminant and ROC analyses were performed with the use of JMP Pro 14 (SAS Institute Inc., Cary, NC, USA).

## 3. Results

### 3.1. Patient Characteristics

In total, 54 patients had 56 liver tumors, 31 tumors were diagnosed as HCC, and 25 tumors were diagnosed as metastatic. Of all the HCCs, 30 were surgically resected and pathologically confirmed, and one was treated by chemotherapy. Of these 30 tumors, eight were well-differentiated HCC, 17 were moderately differentiated HCC, two were poorly differentiated HCC, and two were combined HCC–ICC (intrahepatic cholangiocellular carcinomas). Histological results were unknown in the case of one patient (surgically resected at another hospital) (Table 1). On MR images, 29 tumors showed arterial-phase hyperenhancement, 30 tumors showed nonperipheral washout, and 20 tumors showed enhancing capsules. The etiologies associated with the 31 patients included the following: (a) thirteen patients were diagnosed with hepatitis B virus, (b) seven were diagnosed with hepatitis C virus, (c) two were nonalcoholic steatohepatitis patients, (d) one patient was diagnosed with primary biliary cirrhosis, and (e) eight patients had unknown pathological etiologies.

Of the 25 metastatic tumors, ten were surgically resected and histologically diagnosed as metastatic liver tumors (colorectal cancer, 8; liposarcoma, 1; adrenal cancer, 1), one was diagnosed as metastatic by needle biopsy (primary cancer: neuroendocrine tumor). The remaining 14 tumors were diagnosed based on imaging features and clinical history. Among the eleven histologically diagnosed tumors, nine tumors showed hypovascular and arterial rim-like enhancements (primary cancer: colorectal cancer, 8; adrenal cancer, 1), and two tumors showed hypervascular features (primary cancer: liposarcoma, 1; neuroendocrine tumor, 1). Among the 14 metastatic tumors which were diagnosed based on imaging features and clinical history, all of them showed hypovascular and arterial rim-like enhancements (primary cancer: colorectal cancer, 9; anal cancer, 1; esophageal cancer, 1; ICC, 2; submandibular cancer, 1) (Table 1).

### 3.2. Inter-Reader Reliability

The intraclass correlation coefficients were as follows. Liver parenchyma: sADC = 0.75, ADC = 0.90, MRE = 0.86; HCC; sADC = 0.80, ADC = 0.49, MRE = 0.88; metastases: sADC = 0.95, ADC = 0.97, and MRE = 0.80. These outcomes showed a fair-to-excellent agreement between the two readers.

### 3.3. Diffusion MRI and MRE Shear Modulus

**Figure 3:**
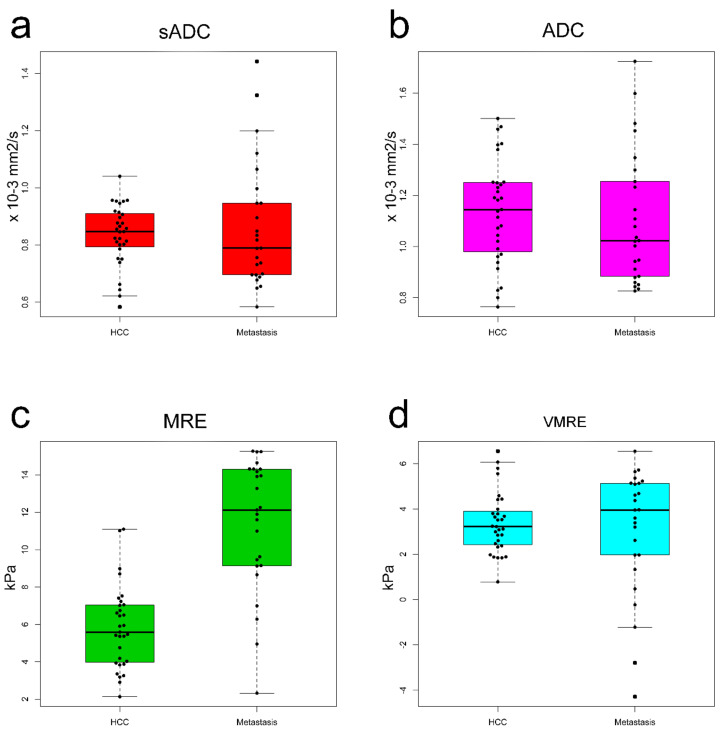
Box plots of ADC values of HCC versus metastasis for (**a**) sADCs and (**b**) ADCs. Mean sADC and ADC values were not significantly different between the HCCs and metastasis. Box plots of the stiffness of the HCCs versus metastasis for (**c**) MRE shear modulus (µ_MRE_) and (**d**) VMRE shear modulus (µ_diff_). The mean metastatic µ_MRE_ value was significantly higher than that of HCCs. The mean µ_diff_ was not significantly different between the HCCs and metastasis.

The ADC values and MRE shear modulus values calculated by the two readers were averaged. The mean ADC value of the HCCs was not statistically different when compared with the mean ADC value of metastases: HCC, 1.14 × 10^−3^ mm^2^/s; metastases, 1.10 × 10^−3^ mm^2^/s (*p* = 0.59). The sADC values, which were obtained at higher *b* values, were significantly lower than the ADC values for all tumors (*p* < 0.001). However, the mean sADC values of the HCCs did not differ significantly compared with that of metastases: HCC, 0.83 × 10^−3^ mm^2^/s; metastases, 0.86 × 10^−3^ mm^2^/s (*p* = 0.56). The mean shear modulus (µ_MRE_) of the HCCs was significantly lower than that of metastases: HCC, 5.83 kPa; metastases, 11.37 kPa (*p* < 0.001) Using parenchyma calibration parameters, the mean virtual shear modulus (µ_diff_) of HCC was not significantly different compared to that of metastasis: HCC, 3.37 kPa; metastases, 3.02 kPa (*p* = 0.56) (Figure 3, Table 3).

### 3.4. Relationships between Shear Modulus (µMRE) and ADC Values (sADC and ADC)

**Figure 4:**
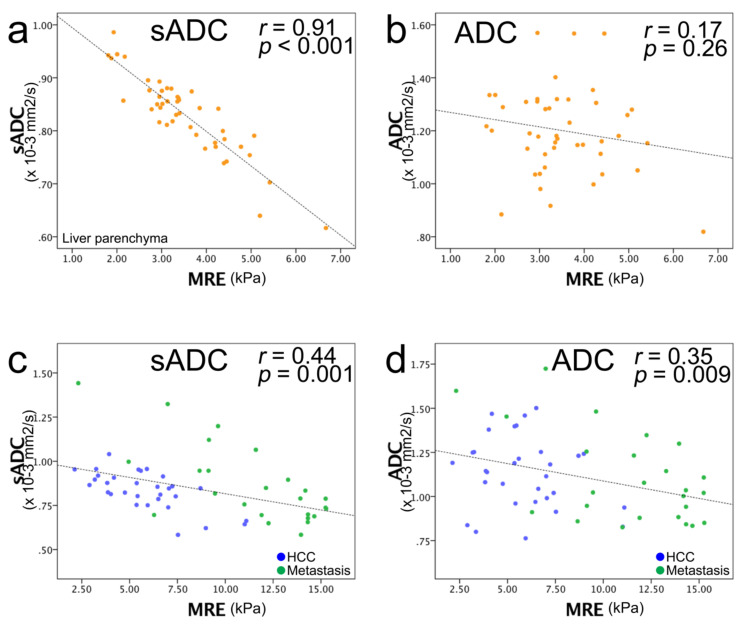

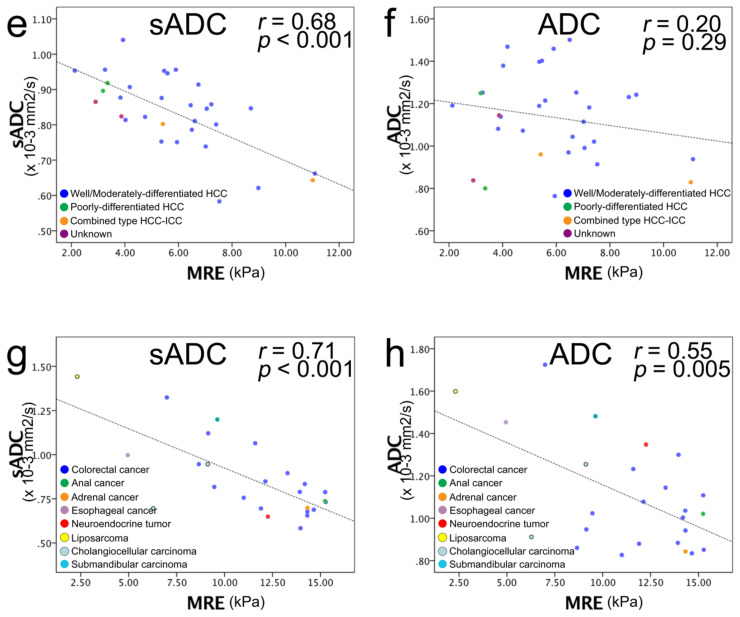
Scatter plots of MRE shear modulus (µ_MRE_; kPa) (horizontal line) versus ADC value (vertical line). (**a**) sADC versus µ_MRE_ of liver parenchyma and (**b**) ADC versus µ_MRE_ of liver parenchyma. (**c**) sADC versus µ_MRE_ of the entire liver tumor and (**d**) ADC versus µ_MRE_ of HCC and metastasis: blue dots, HCC; green dots, metastasis. (**e**) sADC versus µ_MRE_ of HCC, and (**f**) ADC versus µ_MRE_ of HCC: blue dots, well/moderately differentiated HCC; green dots, poorly differentiated HCC; yellow dots, combined type HCC–ICC; purple dots, unknown histology. A strong correlation was observed between sADC and µ_MRE_, and no correlation was observed between ADC and µ_MRE_. (**g**) sADC versus µ_MRE_ of metastasis and (**h**) ADC versus µ_MRE_ of metastasis: blue dots, colorectal cancer; green dots, anal cancer; orange dots, adrenal cancer; purple dots, esophageal cancer; red dots, neuroendocrine tumor; yellow dots, liposarcoma; light blue dots, cholangiocelluar carcinoma; turquoise blue dots, submandibular carcinoma. A strong correlation was observed between sADC and µ_MRE_, and a moderate correlation was observed between ADC and µ_MRE_. The black dashed line corresponds to the linear regression of sADC/ADC versus the MRE values, and not to the VMRE calibration line, which was calculated from the symmetric correlation of the MRE values with ln(S_200_/S_1500_), according to Equation (3).

The correlations between µ_MRE_ and the sADC values were strong and significant for the liver parenchyma, HCCs, and metastases. Specifically, for the liver parenchyma, *r* = 0.91 (*p* < 0.001); malignant tumors, *r* =0.44 (*p* = 0.001); HCC, *r* =0.68 (*p* < 0.001); and for metastases, *r* = 0.71 (*p* < 0.001) (Figure 4). The correlation between µ_MRE_ and ADC was weaker, and significant only for malignant tumors and metastases: liver parenchyma, *r* = 0.17 (*p* = 0.26); malignant tumors, *r* = 0.35 (*p* = 0.009); HCC, *r* = 0.20 (*p* = 0.29); metastases, *r* = 0.55 (*p* = 0.005) (Figure 4).

### 3.5. Virtual Shear Modulus (µ_diff_)

The calibration coefficients α and β of Equation (3) were estimated from a diffusion MRI (VMRE) based on the relationship between µ_MRE_ and sADC. The stiffness values, µ_diff_, were then calculated. The ADC values were not used as the correlation with µ_MRE_ was weaker, as confirmed in an earlier report [1]. The coefficients α and β in the liver parenchyma (α = −9.7 ± 0.7 and β = 13.9 ± 0.7) were close to those reported previously (α = −9.8 ± 0.8, β = 14.0 ± 0.9) (Figure 4a, Table 4) [1,2]. As the tumor’s type was not known at the time of examination, µ_diff_ values were obtained first using the calibration parameters obtained in the parenchyma. With these values, the mean virtual shear modulus (µ_diff_) of HCC was not significantly different compared with that of metastasis (HCC, 3.37 kPa; metastasis, 3.02 kPa, *p* = 0.56) (Figure 3d). Additionally, the virtual shear modulus values were significantly lower than those obtained with MRE (Figure 3c,d), even sometimes negative, implying that the calibration parameters obtained for the parenchyma were not appropriate for the tumors. We then calculated the calibration parameters specifically for each type of tumor: HCC: α = −10.8 ± 2.2 and β = 17.5 ± 2.4; and metastases: α = −8.8 ± 1.8 and β = 21.2 ± 2.1; HCC and metastases: α = −8.1 ± 2.2 and β = 17.2 ± 2.5 (Table 4). The α coefficient in the metastases was higher than that for liver parenchyma but was lower in the case of HCC. The β coefficient was higher in both tumors than in the parenchyma. Using values for α and β in tumors (HCC and metastases) the mean virtual shear modulus (µ_diff_) of HCC was not significantly different compared with that of metastasis: HCC, 8.41 kPa; metastasis, 8.11 kPa (*p* = 0.53). These results were consistent with the fact that there was no significant difference in the mean sADC values between HCC and metastases (Figure 3a).

### 3.6. ROC, Discriminant Analyses and Diagnostic Values

**Figure 5:**
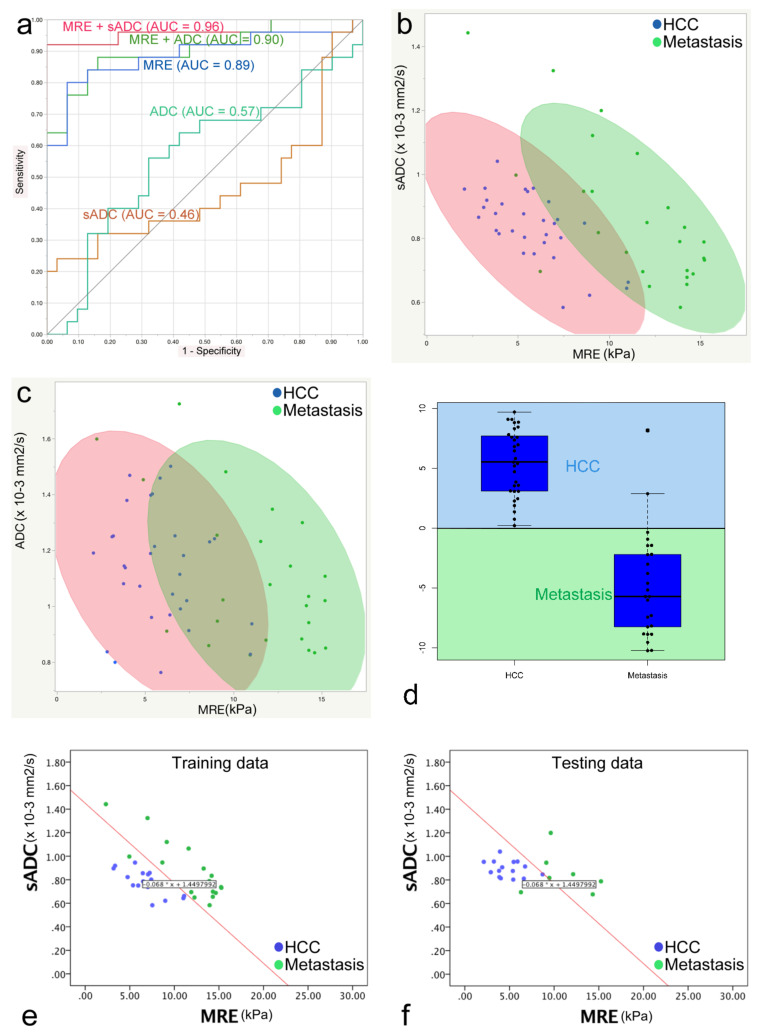
(**a**) Receiver operative characteristic (ROC) analysis used to differentiate HCCs from metastases. The areas under the curve (AUC) of each parameter were as follows: ADC, 0.57; sADC (VMRE), 0.46; MRE, 0.89; MRE and ADC, 0.90; MRE and sADC (VMRE) (from discriminant analysis), 0.96. (**b**) Discriminant analysis to discriminate HCCs and metastases based on the use of µ_MRE_ and sADC values. Blue dots represent HCC, green dots represent metastasis, the red ellipse represents 90% of the area of the HCCs, and the green ellipse represents 90% of the area of the metastases. (**c**) Discriminant analysis to discriminate HCCs and metastases based on the use of µ_MRE_ and ADC values. There is a larger overlap between the two ellipses that use the ADC and MRE shear modulus values compared with the combination of the sADC and MRE shear modulus values. (**d**) Box plots with classification of all tumors using the classifier value (C) obtained with the use of a linear discriminant analysis of the HCC and metastasis training set. A C value > 0 suggests HCC, and C < 0 suggests metastasis. All HCCs were correctly classified (C > 0) and 23 of 25 metastases were correctly classified (C < 0). (**e**,**f**) Linear discriminant analysis to discriminate HCCs and metastases based on the use of the µ_MRE_ and ADC values of the training and testing cohorts. The red line represents the discriminant line between HCC and metastasis calculated from the training cohort data. In the testing cohort, the discriminant line could discriminate HCC from metastasis with high accuracy (HCC, 100%; metastasis, 85.7%).

The following areas under the curve (AUC) values were obtained from the ROC analysis and the discriminant analysis, and were used to differentiate HCC from metastases: ADC, 0.57; sADC, 0.46; MRE, 0.89; MRE and ADC, 0.90; MRE and VMRE (sADC), 0.96 (Figure 5a). Although the combination of MRE and sADC (VMRE) led to a strongest discriminant performance, it did not reach significance (*p* = 0.11). There was also no statistical difference between MRE and MRE + ADC (*p* = 0.42). The cut-off value between HCC and metastases of MRE based on the Youden index was 9.12 kPa. This result prompted us to perform a discriminant analysis which combined VMRE (using sADC) and MRE. From the scatter plots (Figure 5b,d) one can see that the overlap which exists between the tHCC and the metastases for both the sADC and the MRE values can be eliminated when an oblique line of classification is drawn (Figure 5b).

From the training dataset, a linear discriminant analysis yields the following expression for classifier, C,

C = −25.15 sADC (10^−3^ mm^2^/s) − 1.72 µ_MRE_ (kPa) + 36.46 (4)

Lesions were classified as “Metastatic” when C < 0 and as “HCC” when C > 0 (Figure 5d).

By applying classifier C to the testing dataset, the HCCs were completely differentiated (accuracy: 100% 15/15), and the metastases were almost completely differentiated (accuracy: 85.7% 6/7) (Figure 5e,f).

Performances of the classifier C (MRE + VMRE) and MRE alone within the whole cohort in terms of sensitivity, specificity, accuracy, positive predictive value (PPV) and negative predictive value (NPV) for differentiating HCC from metastasis are shown in Table 5. Here also C outperforms MRE alone for all performance markers, but significance was reached only for specificity (*p* = 0.046), probably due to the small size of our cohort.

Such a clear-cut classification, however, could not be established with the ADC values because of a much larger overlap between the two tumor groups (Figure 5c).

## 4. Discussion

MRE was first developed and used for liver fibrosis assessments [11]. Some groups have applied MRE for tumor assessment in the liver [3,4,5,12], as well as in breast and brain tumors [13,14,15]. Hennedige et al. showed a significantly higher accuracy for MRE than DWI in differentiating malignant from benign liver tumors (AUC: MRE = 0.986; DWI = 0.82) [4]. However, a strong correlation between the sADC values and standard MRE stiffness values has been reported in the liver parenchyma [1,2]. In this study, we found strong correlations between the sADC values and the MRE stiffness values not only in the liver parenchyma, but also in liver tumors (liver parenchyma, *r* = 0.91; HCC, *r* = 0.68; metastases, *r* = 0.71), while ADC exhibited no or moderate correlations with the MRE shear modulus (liver, *r* = 0.17; HCC, *r* = 0.20; metastases, *r* = 0.55). While *b* values in the range of 600–1000 s/mm^2^ mainly reflect Gaussian diffusion, the use of higher *b* values, as performed in this study (*b* = 200, 1500 s/mm^2^), increase sensitivity to non-Gaussian diffusion. Therefore, sADC is more sensitive to the tissue microstructure compared with ADC [1]. This surmise may contribute to the good correlation between sADC and MRE. In the liver parenchyma, the calibration parameters which allow the conversion of sADC to VMRE µ_diff_ values were found close to those reported in previous studies with the use of different setups [1,2], and resulted in an accurate estimate of shear stiffness. However, use of these calibration parameters showed that the estimated shear stiffness values in tumors with VMRE were significantly lower than those obtained with MRE. Furthermore, there was no difference in the VMRE values between HCC and metastases, while the MRE values were significantly different. Altogether, these results are consistent with the observation that sADC values were not significantly different between HCCs and metastases. Hence, the calibration parameters between the sADC and MRE stiffness values are different. Nevertheless, the strong correlation observed between the sADC and MRE values in tumors, irrespective of their nature, confirms the existence of an intimate link between tissue shear stiffness as estimated with MRE, and tissue microstructure as evaluated with diffusion MRI, despite the fact that there is currently no biophysical model to bridge these two features and provide adequate calibration parameters α and β for each tumor type. Note that a higher value for α suggests a higher sensitivity of diffusion (sADC) to tissue stiffness, while β is merely a scaling parameter.

The reasons for the aforementioned differences in the calibration parameters between liver parenchyma and tumors, and between tumor types, are expected to be found in the nature of the lesions, their tissue microstructures, and their environment. While liver parenchyma is somewhat homogenous even in the presence of moderate fibrosis, tumor tissues vary considerably according to their nature, especially with metastases, but also in HCC, depending on the tumor stage. The tumor environment may also interfere with the measurements, such as the vascular network surrounding the lesions (23 out of 25 metastases were hypovascular with an arterial rim-like enhancement, 29 of 31 HCCs showed arterial-phase hyperenhancement). While sADC measurements reflect the genuine tissue microstructure within the tumor core, the propagation of MRE shear waves within the tumor may be altered by the surrounding vasculature, thus affecting the estimation of the shear stiffness of the lesion. Indeed, Hennedige et al. found nonsignificant stiffness differences between HCC and metastases [4], while we showed significantly higher metastatic tissue stiffness (11.4 kPa) compared with HCC (5.8 kPa). This discrepancy may also be mainly attributed to the composition differences of primary and metastatic cancers. In our study, 68% of the primary cancers for the metastases were colorectal cancers (other primary cancers in our patient population were liposarcoma, adrenal cancer, and neuroendocrine tumors). Population-based studies have shown that approximately 25–30% of patients diagnosed with colorectal cancer develop liver metastases during the course of their disease, and colorectal cancer is the most common site of liver metastases [16]. Hennedige et al. did not reveal the composition of the primary cancer of the metastases in their study [4]. Metastases from colorectal cancer yielded higher stiffness values because of their histological adenocarcinoma characteristics. In the future, IVIM–based VMRE could provide additional information on tumor heterogeneity that is useful for tumor differentiation or staging [1].

From Figure 5a (ROC analysis) and Figure 5d (discriminant analysis), one can see that a combination of MRE and sADC-based VMRE can almost perfectly discriminate HCCs from metastases (ROC analysis: AUC = 0.96; discriminant analysis classification: HCC = 100%, metastases = 85.7%). These outstanding results suggest that there is some useful classification information in the sADC-based VMRE values, despite the fact that the difference between HCC and metastases is not globally significant. The explanation may be inferred from Figure 5b. One may see that lesions associated with low-MRE values (below 10 kPa) can be classified almost perfectly as HCC or metastases based on their sADC (or resulting VMRE) values (low or high, respectively). Conversely, lesions with high MRE values (above 11 kPa) can be classified almost perfectly as metastases. The global overlap in sADC originates mainly from the lesions with high MRE values observed for the metastases. Although the AUC of MRE + VMRE was higher than for MRE alone, the difference was not significant, probably due to the relatively small size of our cohort. However, specificity of classifier C (92%) to classify HCC over metastases was significantly higher than that of MRE (76%), suggesting that the addition to VMRE to MRE has clinical benefit.

This potentially important finding may be beneficial for many patients because sADC and MRE do not require contrast agents. Differential diagnosis of metastatic liver tumors from primary liver tumors is usually obtained with contrast-enhanced MRI. Recently, imaging techniques have become the mainstay for the assessment of liver lesions, somewhat alleviating the need for biopsy sampling [17]. Besides the invasiveness of biopsy sampling, which is associated with procedural risks and patient discomfort, only limited areas can be sampled [18]. There is a need to achieve a similar level of performance noninvasively. Based on our results, we believe that the combination of sADC and MRE could be an alternative to biopsy sampling.

A clear limitation of our study is a single-center retrospective study with a relatively small sample size. The relationship between sADC and μ_MRE_ must be investigated from a much larger patient cohort with a wider range of liver tumors (including intrahepatic cholangiocarcinoma and benign tumor-like focal nodular hyperplasia, adenoma, etc.). Further work should also aim to improve ROI delineation with the use of tumor segmentation and histograms which VMRE allows to improve accuracy, statistical power, and reduce potential ROI biases. Furthermore, the stiffness of small tumors cannot be measured by MRE owing to its limited spatial resolution. In this study, 13 patients were excluded owing to the MRE image quality.

## 5. Conclusions

In conclusion, the correlation between sADC and the shear modulus (MRE) was high both in the liver parenchyma and in the tumors, despite the fact that the correlation coefficient was lower in liver tumors. The VMRE shear modulus could be accurately estimated in the liver parenchyma, but not in the tumors, as sADC values were not significantly different between HCC from metastases. Compared to MRE only, the combination of MRE and VMRE allowed better discriminant performance between HCC and metastases, probably because of the combined information of stiffness and microstructure.

## Figures and Tables

**Table 1:** Patient demographics.

Parameter	Value		
Number of patients	54		
Men	37		
Women	17		
Mean age (years)	69.4 ± 9.3 years		
Number of liver tumors	56	Histology	Imaging features
HCC ^1^	31	30	1
Type of HCC			
Well differentiated	8		
Moderately differentiated	17		
Poorly differentiated	2		
Combined type HCC–ICC ^2^	2		
Unknown	2		
Metastasis	25	10	15
Primary tumor			
Colorectal cancer	17		
Anal cancer	1		
Esophageal cancer	1		
Adrenal cancer	1		
Neuroendocrine tumor	1		
Liposarcoma	1		
ICC	2		
Submandibular cancer	1		

^1^ HCC: hepatocellular carcinoma; ^2^ ICC: intrahepatic cholangiocellular carcinoma.

**Table 2:** MRI protocol.

**MR-System**	**Discovery 750**		
**MR Sequence**	**MRE**	**DWI (*b* = 0, 800)**	**DWI (*b* = 200, 1500)**
Respiration pattern	Breath-hold	Respiratory-triggered	Breath-hold
Acoustic vibration (Hz)	60	N/A	N/A
TR/TE (msec)	600/62.4	6000–10,000/50.7	3500/60.5
FOV (cm)	42 × 42	36 × 27	36 × 27
Matrix	64 × 64	128 × 128	64 × 64
Thickness (mm)	10	5	7
Slice spacing (mm)	4	2	2
Bandwidth (kHz)	250	250	250
NEX	1	3	1
Acquisition time	14 s	3–4 min	25 s
**MR-System**	**Discovery 750 w**		
**MR Sequence**	**MRE**	**DWI (*b* = 0, 800)**	**DWI (*b* = 200, 1500)**
Respiration pattern	Breath-hold	Respiratory-triggered	Breath-hold
Acoustic vibration (Hz)	60	N/A	N/A
TR/TE (msec)	600/63.4	6000–10,000/63.7	3500/77.9
FOV (cm)	42 × 42	36 × 27	36 × 27
Matrix	64 × 64	128 × 128	64 × 64
Thickness (mm)	10	5	7
Slice spacing (mm)	4	2	2
Bandwidth (kHz)	250	250	250
NEX	1	3	1
Acquisition time	14 s	3–4 min	25 s
**MR-System**	**Signa Architect**		
**MR Sequence**	**MRE**	**DWI (*b* = 0, 800)**	**DWI (*b* = 200, 1500)**
Respiration pattern	Breath-hold	Respiratory-triggered	Breath-hold
Acoustic vibration (Hz)	60	N/A	N/A
TR/TE (msec)	600/63.4	6000–10,000/63.7	3500/77.9
FOV (cm)	42 × 42	36 × 27	36 × 27
Matrix	64 × 64	128 × 128	64 × 64
Thickness (mm)	10	5	7
Slice spacing (mm)	4	2	2
Bandwidth (kHz)	250	250	250
NEX	1	3	1
Acquisition time	14 s	3–4 min	25 s

**Table 3:** Mean apparent diffusion coefficient (ADC) and stiffness (kPa) values of liver tumors.

	*n*	sADC (*b* = 200, 1500 s/mm^2^) Mean ± SD (95% C.I.)	ADC (*b* = 0, 800 s/mm^2^) Mean ± SD (95% C.I.)	MRE (kPa) Mean ± SD (95% C.I.)	VMRE (kPa) Mean ± SD (95% C.I.)
HCC	31	0.83 ± 0.11 (0.80−0.87)	1.14 ± 0.20 (1.06−1.21)	5.83 ± 2.21 (5.02−6.64)	3.37 ± 1.35 (2.88−3.87)
Metastasis	25	0.86 ± 0.22 (0.77−0.95)	1.10 ± 0.26 (1.00−1.21)	11.37 ± 3.54 (9.90−12.83)	3.02 ± 2.79 (1.86−4.17)

**Table 4:** Virtual stiffness calibration parameters (estimated using the whole patient cohort).

µ_diff_ (kPa) = α ln (S_200_/S_1500_) + β		
	α	β
Liver parenchyma	−9.7 ± 0.7	13.9 ± 0.7
HCC and Metastases	−8.1 ± 2.2	17.2 ± 2.5
HCC	−10.8 ± 2.2	17.5 ± 2.4
Metastases	−8.8 ± 1.8	21.2 ± 2.1

**Table 5:** Statistical value from differentiation between HCC and metastasis.

	Sensitivity	Specificity	Accuracy	PPV	NPV
C (cut-off: 0)	100% (31/31)	92% * (23/25)	96.4% (54/56)	93.9% (31/33)	100% (23/23)
MRE (kPa) (cut-off: 9.12 kPa)	93.5% (29/31)	76% * (19/25)	85.7% (48/56)	82.9% (29/35)	90.5% (19/21)

C = classifier (−25.15 sADC − 1.72 MRE + 36.46), MRE = shear modulus calculated by MR elastography; * The difference between C and MRE was significant according to the McNemar’s test (*p* = 0.046).

## Data Availability

The data presented in this study are available on request from the corresponding author. The data are not publicly due to restrictions of privacy.

## References

[1] Le Bihan D., Ichikawa S., Motosugi U. (2017). Diffusion and Intravoxel Incoherent Motion MR Imaging-based Virtual Elastography: A Hypothesis-generating Study in the Liver. Radiology.

[2] Kromrey M.L., Le Bihan D., Ichikawa S., Motosugi U. (2020). Diffusion-weighted MRI-based Virtual Elastography for the Assessment of Liver Fibrosis. Radiology.

[3] Venkatesh S.K., Yin M., Glockner J.F., Takahashi N., Araoz P.A., Talwalkar J.A., Ehman R.L. (2008). MR Elastography of Liver Tumors: Preliminary Results. Am. J. Roentgenol..

[4] Hennedige T.P., Hallinan J.T., Leung F.P., Teo L.L., Iyer S., Wang G., Chang S., Madhavan K.K., Wee A., Venkatesh S.K. (2016). Comparison of magnetic resonance elastography and diffusion-weighted imaging for differentiating benign and malignant liver lesions. Eur. Radiol..

[5] Garteiser P., Doblas S., Daire J.L., Wagner M., Leitao H., Vilgrain V., Sinkus R., Van Beers B.E. (2012). MR elastography of liver tumours: Value of viscoelastic properties for tumour characterisation. Eur. Radiol..

[6] Siegel R.L., Miller K.D., Jemal A. (2020). Cancer statistics, 2020. CA Cancer J. Clin..

[7] Park Y.S., Lee C.H., Kim J.W., Shin S., Park C.M. (2016). Differentiation of hepatocellular carcinoma from its various mimickers in liver magnetic resonance imaging: What are the tips when using hepatocyte-specific agents?. World J. Gastroenterol..

[8] Chernyak V., Fowler K.J., Kamaya A., Kielar A.Z., Elsayes K.M., Bashir M.R., Kono Y., Do R.K., Mitchell D.G., Singal A.G. (2018). Liver Imaging Reporting and Data System (LI-RADS) Version 2018: Imaging of Hepatocellular Carcinoma in At-Risk Patients. Radiology.

[9] Nunn A.D. (2006). The cost of developing imaging agents for routine clinical use. Investig. Radiol..

[10] Guglielmo F.F., Venkatesh S.K., Mitchell D.G. (2019). Liver MR Elastography Technique and Image Interpretation: Pearls and Pitfalls. Radiographics.

[11] Muthupillai R., Lomas D.J., Rossman P.J., Greenleaf J.F., Manduca A., Ehman R.L. (1995). Magnetic resonance elastography by direct visualization of propagating acoustic strain waves. Science.

[12] Thompson S.M., Wang J., Chandan V.S., Glaser K.J., Roberts L.R., Ehman R.L., Venkatesh S.K. (2017). MR elastography of hepatocellular carcinoma: Correlation of tumor stiffness with histopathology features-Preliminary findings. Magn. Reson. Imaging.

[13] McKnight A.L., Kugel J.L., Rossman P.J., Manduca A., Hartmann L.C., Ehman R.L. (2002). MR elastography of breast cancer: Preliminary results. Am. J. Roentgenol..

[14] Sinkus R., Tanter M., Xydeas T., Catheline S., Bercoff J., Fink M. (2005). Viscoelastic shear properties of in vivo breast lesions measured by MR elastography. Magn. Reson. Imaging.

[15] Bunevicius A., Schregel K., Sinkus R., Golby A., Patz S. (2020). REVIEW: MR elastography of brain tumors. Neuroimage Clin..

[16] Hackl C., Neumann P., Gerken M., Loss M., Klinkhammer-Schalke M., Schlitt H.J. (2014). Treatment of colorectal liver metastases in Germany: A ten-year population-based analysis of 5772 cases of primary colorectal adenocarcinoma. BMC Cancer.

[17] Ratziu V., Charlotte F., Heurtier A., Gombert S., Giral P., Bruckert E., Grimaldi A., Capron F., Poynard T. (2005). Sampling variability of liver biopsy in nonalcoholic fatty liver disease. Gastroenterology.

[18] Sagrini E., Renzulli M., Pecorelli A., Stefanini F., Piscaglia F. (2014). Imaging of Liver Tumors in Patients with Chronic Liver Disease. Curr. Radiol. Rep..

